# Clinical safety trial of a thermal jacket among preterm or low birthweight neonates for hypothermia management at a tertiary-level health facility in Bangladesh

**DOI:** 10.7189/jogh.16.04022

**Published:** 2026-02-27

**Authors:** Anisuddin Ahmed, Fariya Rahman, Mohammad Hridoy Patwary, Saifuddin Ahmed, Mohammod Shahidullah, Mats Målqvist, Ahmed Ehsanur Rahman, Shams El Arifeen, Syed Moshfiqur Rahman

**Affiliations:** 1 Global Health and Migration Unit, Department of Women’s and Children’s Health, Uppsala University, Uppsala, Sweden; 2 Maternal and Child Health Division, International Centre for Diarrhoeal Disease Research, Bangladesh (icddr,b), Dhaka, Bangladesh; 3 Department of Population, Family, and Reproductive Health, Bloomberg School of Public Health, Johns Hopkins University, Maryland, USA; 4 Department of Neonatology, Bangladesh Medical University, Dhaka, Bangladesh; 5 Centre for Health and Sustainability, Department of Women’s and Children’s Health, Uppsala University, Uppsala, Sweden

## Abstract

**Background:**

Hypothermia is a common and critical issue for preterm and low birthweight (LBW) neonates, who require effective thermal care management to survive. In a safety trial, we tested a thermal jacket comprising a reusable chemical warming pad and an insulating jacket designed for hypothermia management when skin-to-skin contact is not possible. We assessed its performance in maintaining neonates’ body temperature within the euthermic range (36.5°C to 37.5°C) and evaluated whether it caused any adverse effects.

**Methods:**

We conducted a single-arm, open-label safety trial at a tertiary-level hospital in Bangladesh. We analysed a total of 68 two-hour thermal jacket events involving nine preterm or LBW neonates. The primary outcome was the percentage of events in which the neonate’s body temperature was maintained within the euthermic range for two hours. The secondary outcome was any incidence of adverse clinical signs, including burn, rash, or skin irritation, that we observed during the event. We monitored axillary temperature and other vital signs at the beginning and every 30 minutes throughout each event period. We used a generalised estimating equations-Firth model to assess the effects of study factors on trial outcomes and success status.

**Results:**

The thermal jacket successfully maintained the euthermic temperature range in 96% of events. Its performance remained consistent across varying ambient temperature and humidity conditions, with no significant influence from environmental factors. The three unsuccessful events recorded temperatures outside the euthermic range, with a minimum temperature of 36.3°C and a maximum of 37.8°C. Neonates with initial hypothermia reached euthermia faster and remained euthermic with the support of the thermal jacket. We observed no instances of the above clinical signs.

**Conclusions:**

The thermal jacket safely maintained the preterm or LBW neonate’s body temperature within the euthermic range. Further research is needed to assess the efficacy and effectiveness of this approach in larger clinical settings.

**Registration:**

ClinicalTrials.gov: NCT06277843.

Premature delivery and low birthweight (LBW) are major global health challenges, significantly impacting neonatal mortality and long-term health outcomes. Despite the worldwide prevalence of preterm and LBW births decreasing every year, approximately 13 million neonates are born prematurely (<37 weeks of gestation), and over 20 million neonates are born with LBW (<2500 g) [1,2]. Furthermore, low- and middle-income countries (LMICs) are still facing higher neonatal mortality rates due to LBW and prematurity compared to high-income countries [3]. In Bangladesh, prematurity causes 32% of the total neonatal deaths, with a prevalence of 16.2% [1,4]. Among other major causes, including pneumonia, birth asphyxia, jaundice, respiratory distress, infection, and congenital anomaly, preterm birth and LBW-related complications remain the leading cause of neonatal death worldwide [5].

Hypothermia (body temperature <36.5°C) is a significant concern among preterm and LBW neonates, as their large surface-to-body ratio and high metabolic rate make them more susceptible, contributing to increased mortality rates [6]. Therefore, appropriate thermal care is crucial to support them in attaining and maintaining euthermia (body temperature between 36.5°C and 37.5°C).

Various methods and devices, differing in effectiveness and cost, are available to manage hypothermia, including incubators, radiant warmers, and electric blankets [7]. However, the World Health Organization (WHO) primarily recommends 8–24 hours of kangaroo mother care (KMC) daily, a simple method of caring for preterm and LBW neonates by tying them up to the mother’s chest or a caregiver by the support of a sling, which is proven based on high certainty evidence to be effective for managing hypothermia [8]. This intimate interaction, *i.e.* skin-to-skin contact for 8–24 hours a day, has been found to improve thermal regulation, enhance breastfeeding success, and foster emotional bonding between mothers and their neonates [9]. Moreover, apart from regulating body temperature, this approach reduces the neonates’ risk of mortality and infection and promotes their appropriate growth [8].

Despite its recognised benefits, practice gaps in KMC implementation persist in LMICs, including Bangladesh [10]. While KMC-eligible mothers often express willingness to engage in this practice for the benefit of their neonates, various barriers – including a lack of adequate support from family members, which can hinder mothers’ personal care and rest during their hospital stays, or poorly constructed transportation systems that increase neonates’ risk of hypothermia by preventing mothers from keeping them in the KMC position – impede its consistent application in healthcare settings [11–13]. Moreover, any form of physical discomfort stemming from recent surgical procedures, such as caesarean sections, can further deter mothers from maintaining skin-to-skin practices [14].

To counter these challenges, several thermoregulatory devices have been introduced to support neonatal thermal care, but have demonstrated limited effectiveness in controlled or institutional settings [13,15]. However, their utility in low-resource contexts is often constrained, as most of these devices are expensive and rely on phase-change materials that are limited to obtain due to specialised manufacturing [13,16]. These considerations raise important questions regarding the accessibility and practicality of the existing solutions in contexts like Bangladesh, where economic constraints can limit the effective delivery of healthcare interventions for mothers and their neonates. Consequently, there is a need for an affordable and efficient thermoregulatory device that can offer reliable thermal regulation while facilitating the process of KMC during travel or medical interventions.

In response to these identified needs, we have designed and developed a supplemental thermoregulatory device called the ‘thermal jacket’ comprising a reusable chemical warming pad and an insulating jacket [17], with an aim in maintaining the body temperature of preterm and LBW neonates within the optimal euthermic range between 36.5°C and 37.5°C. In the development phase, we conducted a pre-clinical trial of the thermal jacket on a mannequin in a laboratory setting, and we found that 98% of the events successfully maintained the temperature between 36°C and 38°C for more than two hours [17].

To determine the efficacy and effectiveness of any medical device or treatment through clinical trials among humans, a safety trial (phase I) is essential to identify and mitigate risks [18]. Therefore, building on the results of the laboratory trial, we conducted a clinical safety trial of the thermal jacket among preterm or LBW neonates within a healthcare setting to assess whether the thermal jacket can consistently achieve and maintain the neonate’s body temperature at a euthermic range for a minimum of two hours while observing any potential adverse clinical signs, such as burn, rash, or skin irritation.

## METHODS

### Study design and setting

We conducted a single-arm, open-label trial at Bangladesh Medical University (BMU), a tertiary-level health facility, from June to September 2022, in accordance with international medical device guidelines [19]. Such designs are widely accepted in early-phase device studies, particularly when endpoints such as body temperature can be measured objectively and without bias [20–22].

### Participant screening and enrolment

**Figure 1:**
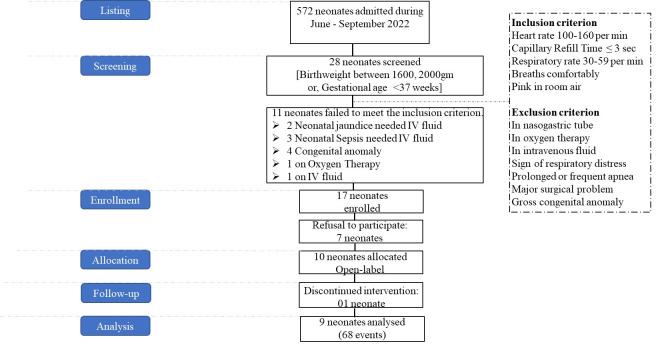
Trial profile.

We held a series of consultative workshops among the neonatologists of BMU and programme implementers of the National Newborn Health Programme (NNHP) of the Directorate General of Health Services under the Ministry of Health and Family Welfare, and adapted the National KMC guideline [23] to set eligibility criteria for screening and enrolment of the neonates (**Figure 1**). For this initial safety trial, we enrolled clinically stable preterm or LBW neonates, and excluded those with acute complications or other conditions requiring concurrent thermal or medical interventions to reduce confounding and minimise clinical risk during early device use.

We enrolled a total of 10 KMC-eligible neonates, either born <37 weeks of gestation or with a birthweight of 1600–2000 g, or both, and conducted 69 distinct events (sample size unit) from them (**Figure 1**). Since we conducted all previous thermal jacket laboratory events on a mannequin for two hours to determine its performance [17], we defined the current events as experimental therapy thermal jacket events on a preterm or LBW neonate, or both, for two hours continuously. Once doctors or nurses identified a neonate eligible for KMC in the perinatal care unit of the Department of Neonatology at BMU, they referred the neonate to the KMC ward within the department. Subsequently, they informed the icddr,b research doctors to help with the enrolment, who then approached the neonate’s legal guardian or caregiver to obtain consent for participation in the safety trial. They counselled the guardians on the importance of KMC practice, including skin-to-skin contact and exclusive breastfeeding, in maintaining thermoregulation for preterm or LBW neonates; explained the use of the thermal jacket and its utilisation to supplement KMC; and finally obtained verbal, followed by written consent.

During enrolment, the neonatal consultant physically re-examined the enrolled neonate. After this, we gave the mother or caregiver the option to place their babies in the thermal jacket at their preferred times during KMC intervals or whenever they felt comfortable. We enrolled each neonate within 24 hours of delivery following the same procedure, and observed them for the next 48 hours. As the chemical warming pad can be reused up to nine times to maintain the temperature range of 36°C to 38°C for two hours [17], we performed a maximum of eight events per neonate during the 48 hours, and again obtained verbal consent from guardians or caregivers before each event. After the first event, we diagnosed one enrolled neonate with jaundice during the routine follow-up and withdrew him from the study for further treatment as per the hospital protocol. We did not include his information in further analysis and results of this paper (**Figure 1**).

During the observation, we continued to conduct routine check-ups by the facility's healthcare providers. At the end of each event, the facility neonatologist thoroughly examined the enrolled neonates and gave final comments on the safety issues of the neonates.

To ensure comprehensive and clear reporting of this study design, implementation, and outcomes, we adhered to the Transparent Reporting of Evaluations with Nonrandomized Design guidelines for transparent reporting of non-randomised evaluations of behavioural and public health interventions [24].

### Thermal jacket intervention procedures and data collection

At the beginning of the event procedure, we recorded neonate’s background characteristics, including perinatal information (birthweight, gestational age, gender, body length, and head circumference), physiological information (age at enrolment, any sign of physical complications), and maternal information (number of antenatal care received, number of parity(s), any antepartum, postpartum or during delivery complications, and mode of delivery). Then, each neonate received the experimental therapy, which involved using the thermal jacket when the mother or caregiver agreed. During each two-hour event, we recorded the axillary temperature of the neonate, along with other vitals such as heart rate, oxygen saturation (SpO_2_) level, and respiratory rate. According to the WHO, axillary temperature measurement is preferred for neonates due to its safety, hygiene, and ease of use [25]. If properly performed, it provides a good approximation of core body temperature. In addition to these data, we observed the development of any adverse clinical signs, including burn, rash or skin irritation. The study nurses conducted all procedures at the beginning and every 30-minute of the two hours under the supervision of the research doctors to maintain close monitoring regarding the safety of the neonate (Figure S1 in the **Online Supplementary Document**). We assessed all 69 events among the 10 enrolled neonates in the same way. During each event, neonates only wore a cap and shocks and were placed on a specific bed in the KMC ward. Furthermore, we also recorded the real-time ambient temperature and humidity during each event. We used a Jitron digital thermometer (measurement accuracy of ±0.1°C) to measure the axillary temperature of the neonates [26], a digital pulse oximeter (Masimo Rad-5V) to record the SpO_2_ level of the neonates, and a LabQuest device to record the temperature and humidity [27]. The research doctors and study nurses received training on the KMC guideline from the national trainers of the NNHP, and on Emergency Triage Assessment and Treatment from the neonatologists of BMU.

### Outcome measures

#### Primary outcome

The primary outcome was the percentage of events in which enrolled neonates maintained an euthermic axillary temperature (36.5°C to 37.5°C) for two hours successfully. We considered an event successful if the neonate’s axillary temperature remained within the euthermic range (36.5°C to 37.5°C) for two hours; or if it started with hypothermia (<36.5°C) and reached the euthermic range at a minimum rate of 0.5°C/h; or if it exceeded or dropped from the range at any time point but returned to it at a minimum rate of 0.5°C/h. We chose this criterion to ensure safety in the context of short-term use, which may differ from definitions in future comparative studies. However, we selected the temperature change and rewarming threshold based on WHO guidelines and prior neonatal hypothermia rewarming literature to ensure physiologically safe temperature rise [28–32].

#### Secondary outcome

The secondary outcome was the occurrence of any adverse clinical signs, including burn, rash, or skin irritation, among the neonates due to exposure to the thermal jacket. Trained doctors and nurses assessed the neonates through clinical observation and documented the findings in a case report form. We did not take photographs due to parental consent limitations and a lack of standardised imaging protocols in the neonatal unit. We chose this approach to minimise handling and distress during this first-in-human testing.

### Sample size calculation and statistical analyses

Based on the laboratory trial [17], we assumed that 98% of the events (with a 5% error margin) would retain the neonate’s body temperature between 36.5°C and 37.5°C for at least two hours with a 99% confidence interval (CI). We calculated that 42 events would be required using the following formula:



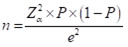



We considered a design effect of 1.15 to account for within-subject correlation due to repeated events in each neonate, and after adjusting for loss to follow-up and/or withdrawal of 20%, we arrived at a sample size of 59 events. Therefore, we enrolled 10 KMC-eligible preterm or LBW neonates to derive the optimum events. Finally, we performed 69 events on 10 KMC-eligible neonates.

We represented the continuous variables in this study through means and 95% CIs, and categorical variables as percentages. We calculated the variability of the sample estimates (proportion or mean) using bootstrapping with 1000 samples to derive a robust CI due to the skewed outcome distribution and small sample size, as conventional t-distribution methods can be inaccurate in such cases [33].

We defined the rate of change (RoC) as the ratio of change in axillary temperature (°C) and the corresponding change in time (minutes). After labelling each event as either successful or failed, we tested the study hypothesis using a one-sided proportion test. We used a χ^2^ test to assess any association between the success status of the event with the neonate and the order of the event or baseline condition, and linear regression to determine the cumulative effect of the baseline temperature among the events. We used the bias-reduced generalised estimating equations (GEE), which combines GEE clustering methods with Firth’s bias reduction to improve parameter estimation in small samples [34]. This GEE-Firth model allowed us to assess the adjusted effect of the study factors on the outcome while accounting for repeated measurements within each neonate, reducing bias from the small sample size, and avoiding problems that occur when certain predictors almost perfectly determine the outcome [35]. Since this model requires balanced data (an equal number of events from each neonate), we selected the first seven events from each neonate to fit the model. We conducted all analyses in *R*, version 4.4.1 (R Core Team, Vienna, Austria).

## RESULTS

Four of the nine neonates were born before 37 weeks of gestation (**Table 1**). Male neonates had higher birthweight, body length, and head circumference than females.

**Table 1:** Perinatal information of the neonates

Neonate	Gender	Gestation age in weeks	Age at enrolment in hours	Birth weight in g	Body length in cm	Head circumference in cm	Birth order
1	Female	36.1	8.1	1825	43	30	1
2	Female	36.3	5.9	1815	40	32	4
3	Female	37.4	9.5	1770	40	31	1
4	Female	38.0	5.7	1890	41	30	3
5	Female	37.3	6.3	1885	41	31	2
6	Male	37.3	6.7	1925	41	31	2
7	Female	37.0	8.5	1770	41	31	4
8	Male	35.6	11.5	1750	38	31	3
9	Male	34.9	9.7	1935	49	31	2

The mean body temperature at each starting point of 68 events for nine neonates was 36.8°C, and it was 37.1°C during the thermal jacket events (intervention) (**Table 2**). On average, the temperature increased 0.3°C during the intervention at each event of all neonates. We recorded hypothermia in a total of 12 (17.6%) out of 68 events at the baseline time point. We observed all the vitals, including x̄ heart rate, respiratory rate, and SpO_2_ levels, to be normal during the intervention [34].

**Table 2:** Physiological responses of the enrolled neonates during events*

Neonate	Number of events	Hypo-thermic at baseline, number of events	Temperature during baseline	Temperature during the intervention	Heart rate during the intervention	Respiratory rate during the intervention	SpO_2_ level during the intervention
1	8	0	36.8 (36.7, 36.9)	37.0 (36.9, 37.0)	133.1 (129.4, 137.0)	47.6 (46.1, 49.2)	94.5 (93.6, 95.3)
2	8	2	36.6 (36.4, 36.8)	36.9 (36.9, 37.0)	125.3 (122.9, 127.8)	43.7 (43.0, 44.6)	96.7 (96.3, 97.2)
3	8	2	37.0 (36.5, 37.5)	37.1 (37.0, 37.2)	124.1 (120.7, 127.6)	42.2 (41.6, 43.0)	96.1 (95.6, 96.7)
4	7	2	36.7 (36.4, 37.0)	37.1 (37.1, 37.2)	126.0 (123.0, 129.0)	43.2 (42.4, 44.1)	95.8 (95.4, 96.3)
5	8	2	36.7 (36.5, 36.9)	36.9 (36.8, 37.1)	122.7 (118.5,127.0)	43.2 (42.6, 44.0)	96.9 (96.5, 97.5)
6	8	2	36.7 (36.3, 37.2)	37.1 (37.0, 37.2)	117.7 (113.6, 121.9)	44.2 (43.4, 45.1)	97.5 (97.0, 98.1)
7	7	0	37.1 (37.0, 37.3)	37.3 (37.2, 37.4)	137.4 (134.3, 140.6)	44.1 (43.4, 44.9)	97.1 (96.5, 97.7)
8	7	0	37.0 (36.9, 37.2)	37.2 (37.1, 37.3)	128.6 (123.7, 133.7)	43.8 (43.2, 44.5)	97.1 (96.6, 97.8)
9	7	2	36.61 (36.2, 37.0)	37.0 (36.9, 37.1)	132.9 (128.2, 137.7)	43.7 (43.1, 44.3)	96.9 (96.3, 97.5)
Overall	68	12	36.81 (36.72, 36.90)	37.07 (37.03, 37.10)	127.0 (125.96, 128.75)	44.0 (43.71, 44.37)	96.50 (96.33, 96.75)

CI – confidence interval, x̄ – mean

*Values presented as x̄ (95% CI) unless specified otherwise.

†Variance not available due to a single observed event.

**Figure 2:**
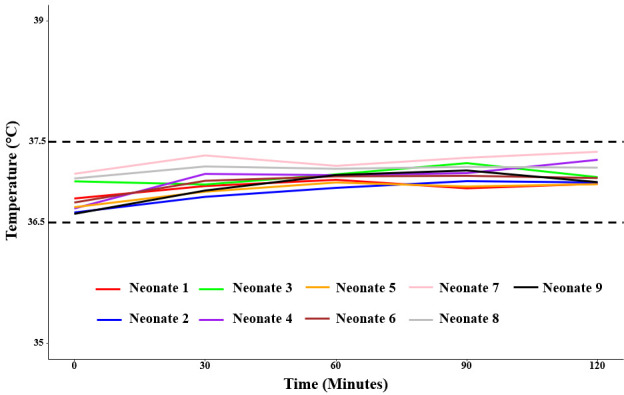
Patterns of the average body temperature of each neonate over different time points between 0 and 120 minutes.

The average body temperature of each enrolled neonate at all events exposed to the thermal jacket between 0 and 120 minutes maintained the range 36.5°C to 37.5°C (**Figure 2**). We observed a consistent pattern of gradual temperature stabilisation, with slight upward trends in some neonates over time (Figure S2 in the **Online Supplementary Document**). The average body temperature of each neonate increased faster between 0 and 30 minutes, stayed steady between 30 and 90 minutes and slowed down between 90 and 120 minutes. Variability in temperature, as indicated by the error bars, remained minimal across all time points, suggesting a stable thermal regulation effect of the jacket. Importantly, no neonate exhibited a rapid increase or decrease in temperature over the event period. These findings suggest that the thermal jacket can successfully maintain euthermia in preterm and LBW neonates without inducing major temperature fluctuations indicative of overheating or hypothermia. Moreover, these RoC were not statistically different between the neonates, as the CIs overlapped for each time interval (Table S1 in the **Online Supplementary Document**).

We observed the RoC in temperature in the first quarter of 120 minutes to be 0.87°C/h for events started with hypothermia, which was higher compared to events started with euthermia (RoC = 0.30°C/h), though there was no significant difference (**Table 3**). We observed a similar pattern among the rest of the quarters of 120 minutes. Also, for both conditions, the RoC in temperature seemed to be reduced as time progressed, which indicates the stability of temperature as it enters the euthermic band.

**Table 3:** Patterns of temperature over time for events that started with hypothermia or euthermia, x̄ (95% CI)

	Temperature			Rate of change in °C/h		
**Time point in minutes**	**Overall (n = 68)**	Hypothermic* (n = 12)	**Euthermic† (n = 56)**	**Overall (n = 68)**	**Hypothermic (n = 12)**	**Euthermic (n = 56)**
0	36.81 (36.72, 36.88)	36.20 (36.07, 36.32)	36.94 (36.88, 37.01)			
30	37.01 (36.93, 37.08)	36.64 (36.42, 36.83)	37.09 (37.03, 37.15)	0.40 (0.19, 0.64)	0.87 (0.35, 1.35)	0.30 (0.11, 0.49)
90	37.10 (37.04, 37.17)	36.88 (36.70, 37.06)	37.15 (37.09, 37.20)	0.09 (−0.01, 0.18)	0.24 (−0.04, 0.54)	0.05 (−0.03, 0.14)
120	37.09 (37.04, 37.16)	36.90 (36.76, 37.08)	37.13 (37.08, 37.18)	−0.01 (−0.19, 0.15)	0.06 (−0.46, 0.60)	−0.03 (−0.19, 0.11)

CI – confidence interval, x̄ – mean

*Hypothermia – temperature less than 36.5°C.

†Euthermia – temperature between 36.5°C and 37.5°C.

Of 68 observed events, 95.6% successfully maintained the body temperature of the neonates in the euthermic range for a continuous two hours. Though the distribution of the success rate differed among the neonates, it didn’t show any significant statistical association with the sequence of the events or baseline condition of the events (**Table 4**). Of the nine enrolled neonates, two had three unsuccessful events with a temperature of 36.3°C (minimum) and 37.8°C (maximum) (Figure S3 in the **Online Supplementary Document**).

**Table 4:** Distribution of success rate over neonate numbers, order of events and baseline temperature of the events

Characteristics	Total events	Successful events in %	*P*-value*
Neonate number			0.09
1	8	100	
2	8	100	
3	8	100	
4	7	100	
5	8	87.5	
6	8	100	
7	7	71.4	
8	7	100	
9	7	100	
Event number			0.19
1	9	100	
2	9	77.8	
3	9	100	
4	9	88.9	
5	9	100	
6	9	100	
7	9	100	
8	5	100	
Baseline temperature of the events			0.47
Euthermia	56	96.4	
Hypothermia	12	91.7	
**Overall**	68	95.6	

*P-value from χ^2^ test.

The adjusted model showed that the success of thermal jacket events in attaining and maintaining the body temperature of the neonates between 36.5°C and 37.5°C for two hours did not depend on the background characteristics of the neonates including gender, gestational age, and age at enrolment and physiological characteristics of the neonates including birthweight, body length, head circumference, heart rate, respiratory rate, and SpO_2_ level (**Table 5**). Moreover, the environmental characteristics, including ambient temperature and humidity, also did not affect the success rate of the thermal jacket events.

**Table 5:** Fitted ‘geefirth’ model for assessing the adjusted effect of the study factors on the maintenance of euthermia.

	Coefficients	SE	*P*-value	95% CI
Heart rate	0.09	0.08	0.28	−0.07, 0.25
Respiratory rate	−0.25	0.47	0.6	−1.17, 0.68
Oxygen saturation (SpO_2_)	−0.52	0.45	0.25	−1.39, 0.35
Gender				
*Girl*				
*Boy*	0.65	0.84	0.44	−0.99, 2.30
Birthweight in gm	0.03	0.02	0.15	−0.01, 0.08
Gestational age in weeks	−1.13	0.85	0.19	−2.79, 0.53
Age at enrolment in hours	0.09	0.07	0.24	−0.06, 0.23
Body length in cm	−0.67	0.62	0.28	−1.88, 0.53
Head circumference in cm	0.71	1.48	0.63	−2.18, 3.61
Ambient temperature in °C	−1.32	1.04	0.21	−3.35, 0.72
Humidity	0.02	0.09	0.84	−0.16, 0.20
Intercept	76.87	0.01	<0.05	76.86, 76.88

CI – confidence interval, SE – standard error

The heart rate of the neonates showed a positive association with euthermia (coefficient = 0.09), but it was not statistically significant (*P*-value = 0.28). Similarly, respiratory rate and SpO_2_ had negative coefficients (−0.25 and −0.52, respectively), suggesting a reduction in the likelihood of maintaining euthermia, but neither effect was significant (*P*-values of 0.60 and 0.25, respectively). We saw a positive association (coefficient = 0.65) of gender (male *vs.* female) with euthermia, although it was not significant (*P*-value = 0.44). Other factors such as birthweight (coefficient = 0.03), gestational age (coefficient = −1.13), body length (coefficient = −0.67), and head circumference (coefficient = 0.71) did not show a statistically significant effect on maintaining euthermia (*P*-values of 0.15, 0.19, 0.28, and 0.63, respectively). We included the age at enrolment in this model (coefficient = 0.09), but also found no significant association (*P*-value = 0.24). Additionally, ambient temperature had a negative coefficient (−1.32; *P*-value = 0.21), suggesting a potential association, although not statistically significant. Humidity had a negligible effect (coefficient = 0.02; *P*-value = 0.84).

We observed no instances of burn, rash, or skin irritation during any thermal jacket event among the neonates (Table S2 in the **Online Supplementary Document**). Moreover, we found no signs of respiratory distress or abnormality of heart rate or SpO_2_ level among the neonates during the jacket events.

## DISCUSSION

The clinical safety trial of the thermal jacket underscores its success in managing hypothermia and ensuring the safety of preterm and LBW neonates in 96% of events. Its results indicate that the thermal jacket is reliable for maintaining euthermia among the preterm and LBW neonates, with a notable success rate in achieving and sustaining the body temperature within the target range of 36.5°C to 37.5°C for at least two hours without any adverse clinical signs, including burn, rash, or skin irritation. In comparison, another trial found a success rate of 67% when testing a similar neonatal thermoregulatory device [15].

The success rate of this trial did not depend on the frequencies of the events. In total, 12 events started with hypothermia (<36.50°C), and the rest started with euthermia. Though the rate of temperature change differed between these two baseline conditions, we did not find any effect of the neonate’s initial body temperature on the jacket’s performance to maintain euthermia for two hours, contrary to findings from a previous study where baseline condition had a significant effect on euthermia maintenance [15].

Among the 4% of unsuccessful events, one started with hypothermia failed to reach euthermia within one hour with a rate of <0.5°C/h. The percentage of the other two events having hyperthermia was less than 3%, which is similar to another safety trial of a neonatal warming device [37]. In two instances, the trial ended with hyperthermia (>37.5°C) and the highest deviation from the euthermia range (36.5°C–37.5°C) we observed was 0.30°C, which is similar to another study [38].

Nearly 18% of the events recorded hypothermia at baseline, underscoring the critical need for effective thermal care interventions immediately after birth [39]. We observed the RoC in temperature at 0.87°C/h for those events in the first 30 minutes, which is a standard thermal care temperature management [40]. Moreover, according to Fourier’s law of heat conduction, heat transfer between two objects depends on their temperature difference [41] – the larger the temperature difference, the faster the rate of temperature change. We observed this behaviour in this event; the rate of temperature change differed between the two baseline conditions of the events, where hypothermic events exhibited a higher initial RoC in temperature compared to euthermic ones. Additionally, the RoC decreases as the temperature reaches the euthermic range. This difference could suggest that the thermal jacket may be particularly beneficial for neonates having hypothermia, reinforcing the argument for early and proactive thermal care management as part of standard care protocols [7]. Notably, the baseline temperature of the consecutive events gradually increased with repeated thermal jacket use (Table S3 in the **Online Supplementary Document**). Linear regression showed a significant positive association (*β* = 0.0503; *P*-value <0.001), indicating a cumulative effect, with a 0.05°C rise per event. This suggests that repeated use of the thermal jacket may enhance thermal stability in neonates over time, potentially reducing the risk of hypothermia.

The observed neonate’s body temperature increases 0.3°C on average while wearing the jacket, showing the thermal jacket's potential for providing rapid and reliable thermoregulatory support. The pattern of temperature maintenance exhibited a plateau, suggesting that the device stabilises the thermal environment for the neonates after initial fluctuations, which is a desirable characteristic for any thermal care management strategy [42].

Though the literature suggests that a large head circumference and smaller body length are associated with hypothermia due to an increased risk of heat loss, we did not find any significant effect of these factors on the thermal jacket’s performance in maintaining euthermia. Similarly, neither neonatal background characteristics, including age at enrolment, gender, and gestational age, nor physiological characteristics, including birthweight, heart rate, respiratory rate, and SpO_2_ levels, impacted the device's success in maintaining euthermia of the neonates, which is comparable to the performance of another warming device [15]. This indicates the robustness of the thermal jacket across a diverse range of neonate profiles and gestures towards the universality of its application, thereby accommodating varying clinical situations that healthcare providers may encounter.

In general, thermal injuries to living tissues occur as a function of temperature and duration of exposure to a heat source [43]. A surface temperature of 42°C to 43°C causes a second-degree burn on human skin after a contact period of 12 to 20 hours [43]. However, one study reported that a neonate might get burned if it is exposed to any warming devices whose maximum temperature exceeds 42°C (107.6°F) [44]. In our study, though in a few instances, the thermal jacket failed to maintain the desired temperature range of the neonates, but it did not go above 38.0°C at any point; therefore, using the thermal jacket will have no possibility of burning the neonate's skin (Figure S3 in the **Online Supplementary Document**). Moreover, the vitals (heart rate; repiratory rate; SpO_2_) also did not show any abnormality. We recorded no instances of adverse clinical signs, such as burn, rash, or skin irritation due to exposure to the thermal jacket, similar to findings in other studies [15,37].

### Strengths

The number of events has enough power to capture the 98% success rate while adjusting for the within-neonate correlation. Though the thermal jacket showed a 96% success rate in maintaining the euthermia of the neonates, the hypothesised success rate (98%) was not significantly different from the observed success rate (*P*-value = 0.16). The bootstrap 95% CI was 89.70%, 100.00%, with a point estimate of 95.57% for the success rate, also indicating that the observed success rate was not different from the hypothesised success rate. The bootstrap CI also showed the variation in the success rate that might have happened if we observed a different cohort of events. Moreover, to evaluate the safety of the thermal jacket during event, we found that handwashing with detergent is enough to clean the jacket, as evident in our virology report, which suggests no existence of microorganism, including *Klebsiella pneumoniae*, *Escherichia coli*, and so on [17].

### Limitations

Due to time constraints, we could not observe the effect of seasonal variation on the performance of the thermal jacket. Also, due to ethical concerns, we used a non-random selection of the study participants [20]. We recognise that the absence of a comparative control group limits direct attribution of outcomes to the thermal jacket. However, regulatory standards for medical device evaluation specifically acknowledge that single-arm designs are appropriate for initial safety assessments when the primary endpoints are objective [20]. In particular, the UK Medicines and Healthcare products Regulatory Agency guidance on statistical considerations for clinical investigations of medical devices highlights that the majority of device studies do not require a comparative group, and that a single-arm design may be sufficient to achieve study objectives when endpoints can be measured objectively [22]. Similarly, ISO 14155:2020, the international standard for clinical investigation of medical devices, explicitly recognises single-arm methodologies as valid approaches for initial safety assessment [19]. Since we excluded neonates with common comorbidities, our study population represents an idealised, relatively stable subgroup. Although this limits generalisability to the broader LMIC neonatal population, we emphasised the safety while generating reliable and clinically meaningful evidence before proceeding to larger events. The later phases of these clinical trials would be conducted with diverse and high-risk neonate populations. Besides, our safety assessment relied on visual inspection without standardised scoring (*e.g.* Neonatal Skin Condition Score [45]) or photographic documentation, which introduces subjectivity and may miss subtle or subclinical skin changes. According to the guidelines provided by the World Health Organization and the Association of Women’s Health, Obstetric and Neonatal Nurses, monitoring body temperature to detect early thermal injury or overheating before it appears on the skin is preferable [26,46]. Moreover, this trial was not powered to detect sex-based differences in thermal outcomes or safety trials. Considering sex and gender is essential in neonatal thermal care research, as physiological responses may vary, future studies could explore these differences to inform personalised approaches and ensure equitable care.

However, findings from this clinical safety trial of the thermal jacket are promising and could contribute to the ongoing discourse about thermal care management in neonatal care, especially in low-resource settings, such as Bangladesh. This capability is particularly significant given the high incidence of hypothermia among preterm and LBW neonates, which significantly contributes to neonatal mortality and morbidity.

## CONCLUSIONS

The thermal jacket could be a potential thermoregulatory device for thermal care management of preterm or LBW neonates. The thermal jacket successfully maintained the neonate’s body temperature within the euthermic band for two hours and showed no incidence of adverse health outcomes. Further large-scale efficacy and effectiveness trials with a robust study design remain essential for the successful implementation of the thermal jacket in the existing healthcare settings in Bangladesh.

## Additional material


Online Supplementary Document
