# Supplementary material for: Clinical safety trial of a thermal jacket among preterm or low birthweight neonates for hypothermia management at a tertiary-level health facility in Bangladesh
Source: J Glob Health. 2026 Feb 27;16:04022. doi: 10.7189/jogh.16.04022 (PMC12945343; doi:10.7189/jogh.16.04022)
Supplement: Online Supplementary Document [file jogh-16-04022-s001.pdf]

Supplement to: Ahmed A, Rahman F, Patwary MH, Ahmed S, Shahidullah M, Målqvist M, Rahman AE, Arifeen SE, Rahman SM. Clinical safety trial of a thermal jacket among preterm or low birthweight neonates for hypothermia management at a tertiary-level health facility in Bangladesh. J Glob Health. 2026;16:04022.

Table S1. Rate of change of body temperature (mean temperature [95% CI]) over time for each neonate

	Rate of change [mean (95% CI)]		
**Neonate**	**Time 30 – Time 0**	**Time 90 – Time 30**	**Time 120 – Time 90**
Neonate 1	0.15 [-0.05, 0.72]	-0.03 [-0.24, 0.17]	0.06 [-0.14, 0.39]
Neonate 2	0.19 [-0.06, 0.86]	0.19 [-0.01, 0.42]	-0.02 [-0.52, 0.38]
Neonate 3	-0.04 [-1.11, 1.04]	0.26 [-0.14, 0.69]	-0.17 [-0.89, 0.16]
Neonate 4	0.43 [0.38, 1.35]	0.01 [-0.19, 0.20]	0.17 [-0.21, 0.79]
Neonate 5	0.19 [-0.18, 0.95]	0.06 [-0.34, 0.41]	0.03 [-0.52, 0.78]
Neonate 6	0.27 [-0.17, 1.32]	0.06 [-0.20, 0.36]	-0.03 [-0.53, 0.46]
Neonate 7	0.22 [-0.06, 0.86]	-0.03 [-0.30, 0.25]	0.07 [-0.32, 0.67]
Neonate 8	0.15 [-0.20, 0.77]	-0.00 [-0.28, 0.25]	-0.01 [-0.36, 0.40]
Neonate 9	0.29 [-0.10, 1.32]	0.25 [-0.01, 0.49]	-0.14 [-0.90, 0.25]
Overall	0.20 [0.14, 0.63]	0.08 [-0.01, 0.18]	-0.01 [-0.18, 0.15]

Table S2. Status of adverse clinical signs, including burn, rash, or skin irritation during each event

	Event 1	Event 2	Event 3	Event 4	Event 5	Event 6	Event 7	Event 8
Neonate 1	✖	✖	✖	✖	✖	✖	✖	✖
Neonate 2	✖	✖	✖	✖	✖	✖	✖	✖
Neonate 3	✖	✖	✖	✖	✖	✖	✖	✖
Neonate 4	✖	✖	✖	✖	✖	✖	✖	✖
Neonate 5	✖	✖	✖	✖	✖	✖	✖	✖
Neonate 6	✖	✖	✖	✖	✖	✖	✖	✖
Neonate 7	✖	✖	✖	✖	✖	✖	✖	✖
Neonate 8	✖	✖	✖	✖	✖	✖	✖	✖
Neonate 9	✖	✖	✖	✖	✖	✖	✖	✖

Green: No adverse effect; Red: Skin burn; Yellow: Skin irritation; Orange: Skin rash

Table S3. Baseline temperatures of the 68 events across nine neonates

Id/Event	1	2	3	4	5	6	7	8	Total
**Neonate 1**	36.8	36.7	36.8	36.7	37.0	37.1	36.7	36.7	36.8
**Neonate 2**	36.4	36.6	36.5	36.3	36.7	36.7	36.9	36.9	36.6
**Neonate 3**	35.9	37.1	36.4	37.2	37.3	37.4	37.2	37.5	37.0
**Neonate 4**	36.6	36.9	36.3	36.5	36.8	37.2	36.3	NA	36.7
**Neonate 5**	36.3	36.4	36.7	36.9	36.9	36.6	36.7	37.1	36.7
**Neonate 6**	35.7	36.2	37.0	37.1	36.9	37.0	37.1	37.0	36.8
**Neonate 7**	37.2	37.1	37.3	37.1	37.1	36.8	37.1	NA	37.1
**Neonate 8**	37.2	36.8	37.1	37.2	37.3	37.0	36.8	NA	37.0
**Neonate 9**	36.0	36.1	37.0	36.9	36.8	36.6	36.8	NA	36.6
**Total**	**36.5**	**36.7**	**36.8**	**36.9**	**37.0**	**36.9**	**36.8**	**37.1**	**36.8**

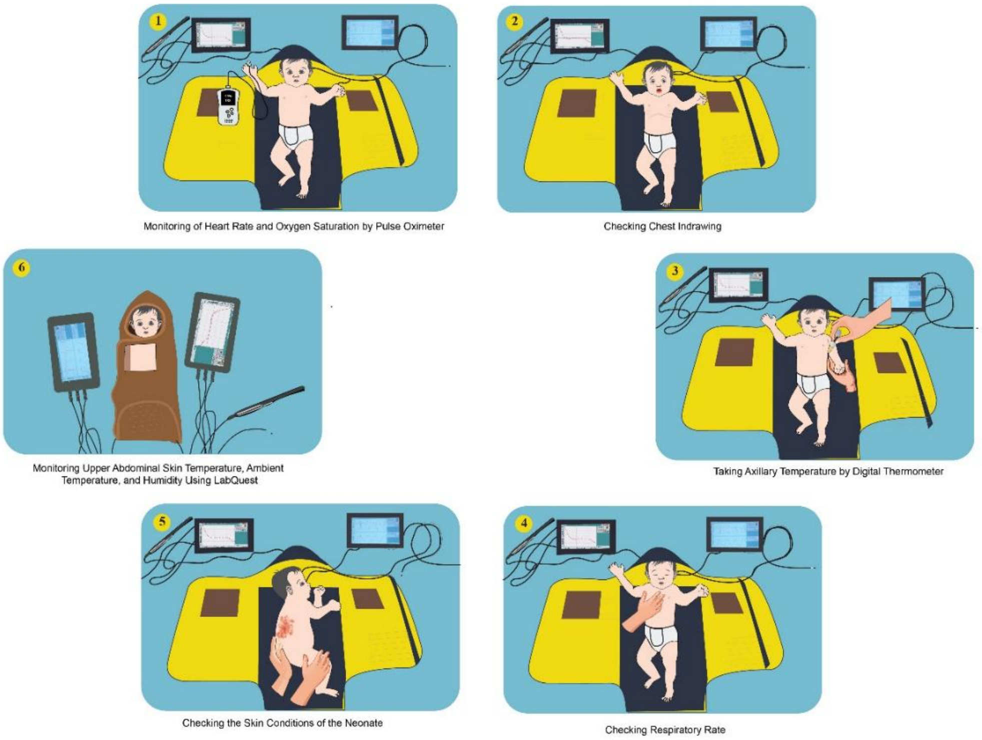

Figure S1. General examinations during intervention (Thermal Jacket)

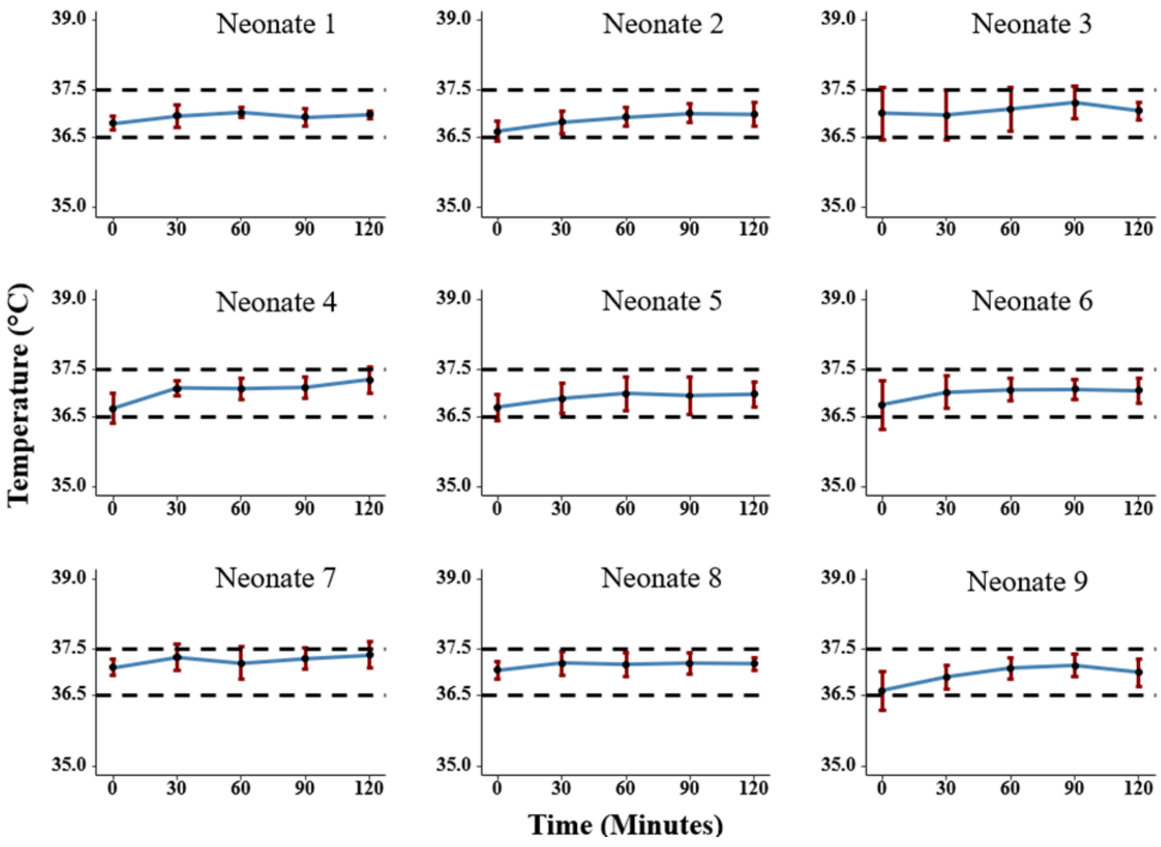

Figure S2. The average temperature of each neonate throughout the events

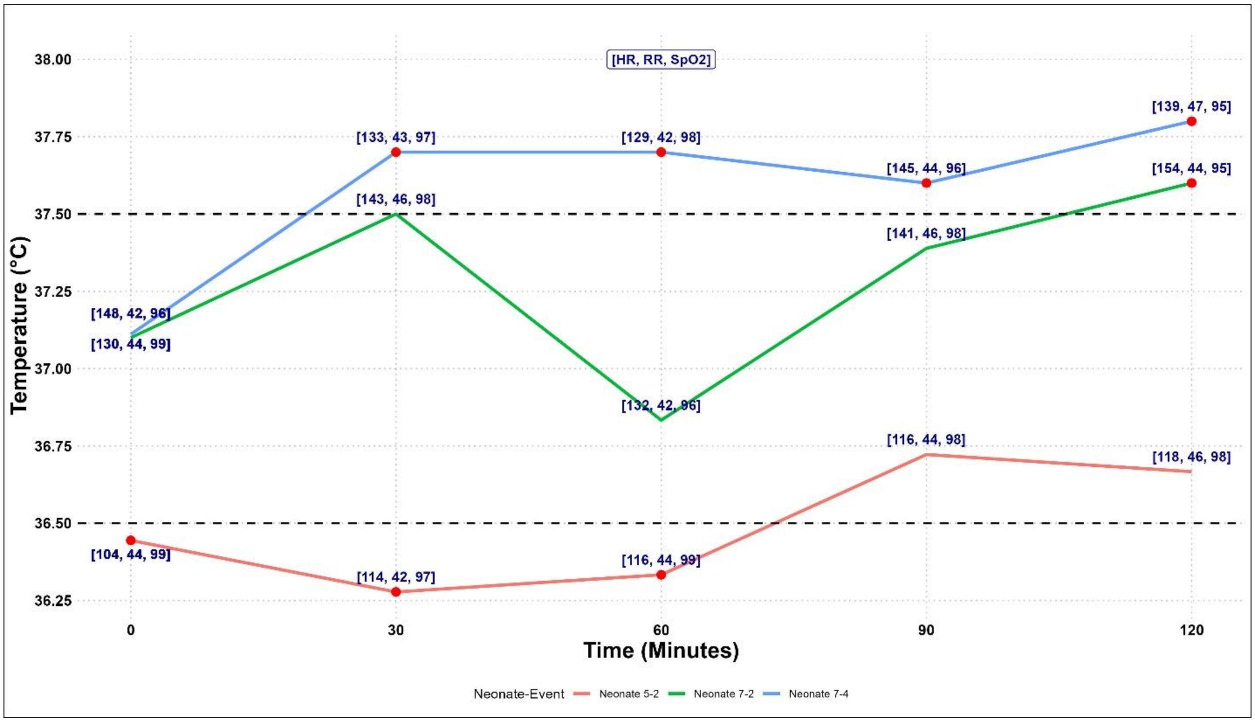

Figure S3. Patterns of the neonate's body temperature along with vitals (HR; RR; SpO_2) for three unsuccessful events.
